# Supplementary material for: Mass distribution of magnetized quark-nugget dark matter and comparison with requirements and observations
Source: Sci Rep. 2020 Oct 21;10:17903. doi: 10.1038/s41598-020-74984-z (PMC7578839; doi:10.1038/s41598-020-74984-z)
Supplement: Supplementary file 1 — Supplementary Information. [file 41598_2020_74984_MOESM1_ESM.pdf]

Supplementary Information for

Mass distribution of magnetized quark-nugget dark matter and comparison with requirements and observations

J. Pace VanDevender, Ian Shoemaker, T. Sloan, Aaron P. VanDevender, Benjamin A. Ulmen

Supplementary Note: Quark-nugget research summary

Macroscopic quark nuggets [7] are also called strangelets [8], nuclearites [9], AQNs [10], slets [11], and Macros [12]. The theory of quark nuggets by Witten [7] indicates quark-nuggets are in an ultra-dense, color-flavor-locked (CFL) phase [18] of quark matter. Steiner, *et al.* [19] showed that the ground state of the CFL phase is color neutral and that color neutrality forces electric charge neutrality, which minimizes electromagnetic emissions. However, Xia, *et al.* [11] found that quark depletion causes the ratio Q/A of electric charge Q to baryon number A to be non-zero and varying at $Q/A \sim 0.32\ A^{-1/3}$ for $3 < A < 10^5$. In addition to this core charge, they find that there is a large surface charge and a neutralizing cloud of charge to give a net zero electric charge for sufficiently large A. So quark nuggets with $A \gg 1$ are both dark and very difficult to detect with astrophysical observations.

Witten and Xia, *et al.* also showed their density should be somewhat larger than the density of nuclei, and their mass very large, even the mass of a star. Large quark nuggets are predicted to be stable [7, 8, 18, 20] with mass between 10^{-8} kg and 10^{20} kg within a plausible but uncertain range of assumed parameters of quantum chromodynamics (QCD) and the MIT bag model with its inherent limitations [21].

Although Witten assumed a first-order phase transition formed quark nuggets, Aoki, *et al.*[22] showed that the finite-temperature QCD transition that formed quark nuggets in the hot early universe was very likely an analytic crossover, involving a rapid change as the temperature varied, but not a real phase transition. Recent simulations by T. Bhattacharya, *et al*. [23] support the crossover process.

A combination of quark nuggets and anti-quark nuggets have also been proposed within constraints imposed by observations of neutrino flux [24]. Zhitnitsky [10] proposed that Axion Quark Nuggets (AQN) that forms quark and anti-quark nuggets generated by the collapse of the axion domain wall network. Although the model relies on the hypothetical particle that is a proposed extension of the Standard Model to explain CP violation, it appears to explain a wide variety of longstanding problems and leads to quark and anti-quark nuggets with a narrow mass distribution at ~1 kg [25]. Atreya, *et al*. [26] also found that CP-violating quark and anti-quark scatterings from moving Z(3) domain walls should form quark and anti-quark nuggets, regardless of the order of the quark-hadron phase transition.

Experiments by A. Bazavov, *et al*. [27] at the Relativistic Heavy Ion Collider (RHIC) have provided the first indirect evidence of strange baryonic matter. Additional experiments at RHIC may determine whether the process is a first order phase transition or the crossover process. In either case, quark nuggets could have theoretically formed in the early universe.

In 2001, Wandelt, *et al*. [13] showed that quark nuggets meet all the theoretical requirements for dark matter and are not excluded by observations when the stopping power for quark nuggets in

the materials covering a detector is properly considered and when the average mass is >10^5 GeV (~2×10^{-22} kg). In 2014, Tulin [15] surveyed additional simulations of increasing sophistication and updated the results of Wandelt, *et al*. The combined results help establish the allowed range and velocity dependence of the strength parameter and strengthen the case for quark nuggets. In 2015, Burdin, *et al.* [28] examined all non-accelerator candidates for stable dark matter and also concluded that quark nuggets meet the requirements for dark matter and have not been excluded experimentally. Jacobs, Starkman, and Lynn [12] found that combined Earth-based, astrophysical, and cosmological observations still allow quark nuggets of mass 0.055 to 10^{14} kg and 2×10^{17} to 4×10^{21} kg to contribute substantially to dark matter. The large mass means the number per unit volume of space is small, so detecting them requires a very large-area detector.

These studies did not consider an intrinsic magnetic field within quark nuggets. However, Tatsumi [16] has shown that the lowest-energy configuration of a quark nugget is a ferromagnetic liquid held together by strong nuclear forces. He calculates the value of the magnetic field at the surface of a quark-nugget core inside a magnetar to be $10^{12\pm1}$ T, which is large compared to expected values for the magnetic field at the surface of a magnetar star with a quark-nugget core. For a quark nugget of radius r_{QN} and a magnetar of radius r_s, the magnetic field scales as $(r_{QN}/r_s)^3$. Therefore, the surface magnetic field of a magnetar is smaller than 10^{12} T because $r_s > r_{QN}$. Since quark-nugget dark matter is bare, the surface magnetic field of what we wish to detect is $10^{12\pm1}$ T.

Although the cross section for interacting with dense matter is greatly enhanced [17] by the magnetic field which falls off as radius r_{QN}^{-3}, the collision cross section is still many orders of magnitude too small to violate the collision requirements [12, 13, 15, 28] for dark matter and will be discussed below.

Chakrabarty [29] showed that the stability of quark nuggets increases with increasing magnetic field ≤ 10^{16} T, so the large self-field described by Tatsumi should enhance their stability. Ping, *et al.*[30] showed that magnetized quark nuggets should be absolutely stable with the newly-developed equivparticle model, so the large self-field described by Tatsumi should ensure that they will not decay during the aggregation process examined in this paper.

The large magnetic field also alters MQN interaction with each other through magnetic attraction and enhances their interaction with ordinary matter through the greatly-enhanced stopping power of the magnetopause around high-velocity MQNs moving through a plasma [17]. Searches [31] for quark nuggets with underground detectors would not be sensitive to highly magnetic ones. For example, the paper by Gorham and Rotter [24] about constraints on anti-quark nugget dark matter (which do not constrain quark-nuggets unless the ratio of anti-quark nuggets to quark nuggets is shown to be large) assumes that limits on the flux of magnetic monopoles from analysis by Price, *et al.* [32] of geologic mica buried under 3 km of rock are also applicable to quark nuggets. Gorham and Rotter also cite work by Porter, *et al.* [33-34] as constraining quark-nugget (nuclearite) contributions to dark matter by the absence of meteor-like objects that are fast enough to be quark nuggets. Bassan, *et al*. [35] looked for quark nuggets (nuclearites) with gravitational wave detectors and found signals much less than expected for the flux of dark matter. However, all of these experiments assumed the cross section for momentum transfer is the geometric cross section, which is many orders of magnitude smaller than the cross section of its magnetopause [17].
